# Dexamethasone in the era of COVID-19: friend or foe? An essay on the effects of dexamethasone and the potential risks of its inadvertent use in patients with diabetes

**DOI:** 10.1186/s13098-020-00583-7

**Published:** 2020-09-07

**Authors:** Janine Alessi, Giovana B. de Oliveira, Beatriz D. Schaan, Gabriela H. Telo

**Affiliations:** 1 Medical Science Program: Endocrinology, Universidade Federal do Rio Grande do Sul, Porto Alegre, Brazil; 2 Internal Medicine Department, Hospital São Lucas-Pontifícia Universidade Católica do Rio Grande do Sul, Porto Alegre, Brazil; 3 School of Medicine, Pontifícia Universidade Católica do Rio Grande do Sul, Porto Alegre, Brazil; 4 School of Medicine, Universidade Federal do Rio Grande do Sul, Porto Alegre, Brazil; 5 Endocrinology Division, Hospital de Clínicas de Porto Alegre, Porto Alegre, Brazil; 6 National Institute of Science and Technology for Health Technology Assessment (IATS), CNPq, Porto Alegre, Brazil; 7 Medicine and Health Sciences Program, Pontifícia Universidade Católica do Rio Grande do Sul, Porto Alegre, Brazil

**Keywords:** Corticosteroids, Diabetes mellitus, COVID-19 pandemic, Metabolic effects, Dexamethasone

## Abstract

**Background:**

The disclosure in the media of a benefit with the use of dexamethasone in patients with COVID-19 infection sets precedents for self-medication and inappropriate use of corticosteroids.

**Methods:**

This is a critical interpretive synthesis of the data available in the literature on the effects of the use of corticosteroids and the impact that their indiscriminate use may have on patients with diabetes. Reviews and observational and experimental studies published until June 18, 2020 were selected.

**Results:**

Corticosteroids are substances derived from cholesterol metabolism that interfere with multiple aspects of glucose homeostasis. Interactions between corticoid receptors and target genes seem to be among the mechanisms responsible for the critical functions of glucocorticoids for survival and anti-inflammatory effects observed with these medications. Corticosteroids increase hepatic gluconeogenesis, reduce peripheral use of glucose and increase insulin levels. Previous studies have shown that glucocorticoids have a pro-adipogenic function, increasing deposition of abdominal fat, and lead to glucose intolerance and hypertriglyceridemia. In addition, these drugs play a role in controlling liver metabolism and can lead to the development of hepatic steatosis. Glucocorticoids reduce the recruitment of osteoblasts and increase the number of osteoclasts, which results in increased bone resorption and greater bone fragility. Moreover, these medications cause water and sodium retention and increase the response to circulating vasoconstrictors, which results in increased blood pressure levels. Chronic or high-dose use of corticosteroids can, by itself, lead to the onset of diabetes. For those who were already diagnosed with diabetes, studies show that chronic use of corticosteroids leads to a 94% higher risk of hospitalization due to diabetes complications. In addition to the direct effects on glycemic control, the effects on arterial pressure control, lipids and bone metabolism also have a potential for severe consequences in patients with diabetes.

**Conclusion:**

Fear and uncertainty toward a potentially serious infection may lead people to self-medication and the inappropriate and abusive use of corticosteroids. More than ever, it is necessary for health professionals to be alert and able to predict damages related to the use of these drugs, which is the first step to minimize the potential damages to come.

## Background

The saga in search of a drug capable of changing the natural history of the coronavirus disease 19 (COVID-19) experienced a new chapter: dexamethasone emerges with the aim of reducing mortality in hospitalized patients. However, before its emergence, other treatments arrived with the promise of miraculous achievements and stayed behind, remaining only adverse effects and lack of medications access for clinical conditions that would truly benefit from their use. In this context, the dimensions of self-medication and drug misuse are currently alarming. An example of this is hydroxychloroquine: in the same week of the release of a small study that showed a potential effect on reducing the viral load of infected patients, the drug’s stocks ran out in pharmacies in Brazil, putting patients with lupus and rheumatoid arthritis at risk because of its unavailability [1, 2]. Although subsequent studies refuted the benefits of its use, the consumption of hydroxychloroquine in Brazil remained high. In the first week of June 2020, the country imported more than two million doses of this drug despite all recommendations against its routine use from international organizations and scientific community [3–5]. Similar expectations were experienced by azithromycin, ivermectin and, now, dexamethasone.

In the past few days, promising results from the use of dexamethasone in patients with COVID-19 infection have been published. The results of the RECOVERY study (Randomised Evaluation of COVID-19 Therapy) showed a potential reduction in mortality of up to one-third (29.3% vs. 41.4%; rate ratio, 0.64; 95% CI 0.51 to 0.81) in patients on mechanical ventilation and up to one-fifth (23.3% vs. 26.2%; rate ratio, 0.82; 95% CI 0.72 to 0.94) in patients using oxygen [6]. The rapid dissemination through the media of the effects of dexamethasone opens the door to the indiscriminate use of this medication and puts us on the brink of an unprecedented clinical collapse. For professionals who care for patients with diabetes mellitus, more than ever it is necessary to keep in mind the impact that the use of corticosteroids may have on glycemic control and other metabolic parameters. Moreover, individuals with obesity, insulin resistance and pre-diabetes may experience the change of their disease state to overt diabetes by the use of these drugs.

Training health professionals to predict damages related to the use of these drugs and to publicize the need for cautious and rational use of dexamethasone is the first step to minimize the potential damages to come. Based on this, the present study aimed to review the mechanism of action of corticosteroids, their metabolic effects and the possible consequences of their indiscriminate use, especially in patients with diabetes. A practical summarized guide of considerations prior to its use, treatment of exacerbated hyperglycemia and strategies for corticosteroids withdrawal will be provided.

## Methods

This is a critical interpretive synthesis of the data available in the literature on the effects of the use of corticosteroids, potential risks and benefits of their administration and the impact that their indiscriminate use may have on patients with diabetes. In addition, practical issues on glucocorticoid use and withdrawal in diabetes are also provided. An iterative search strategy was carried out, with a review of articles indexed on the PUBMED platform, as well as a manual review based on the main articles’ reference list when appropriate. The key words for the search were “corticosteroids AND mechanism of action”, “corticosteroids AND pharmacology”, “corticosteroids AND diabetes”, “corticosteroids AND COVID-19”, “corticosteroids AND hyperglycemic AND treatment”, “corticosteroids AND withdrawal”. Reviews and observational and experimental studies in vitro and in vivo, including either animals or humans, published until June 18, 2020, addressing aspects related to the effects of the use of corticosteroids were selected. A screening based on the title and abstract of the articles that would be included in the analysis was carried out during the iterative search. After that, the full text was read. The research was conducted by two independent researchers (J.A. and G.B.O.), who were responsible for reviewing specific content and synthesizing the information considered relevant in order to offer an overview on the subject and discuss its insertion in the current context. All authors reviewed and agreed with the final content of the synthesis.

### The role of corticosteroids

Corticosteroids are substances derived from cholesterol metabolism that share three 6-carbon hexane rings and one 5-carbon pentane ring in their structure. The term corticosteroid is used clinically to describe agents with glucocorticoid activity and includes molecules that have two carbons at position 17 on the pentane ring and methyl groups at the carbon position 18 and 19 [7]. Cortisol, which is an endogenous glucocorticoid, is produced by the adrenal cortex and its release depends on the proper functioning of the hypothalamic–pituitary–adrenal axis, which is essential for the maintenance of vital functions. Under non-stressed conditions, the human body produces approximately 20 mg of cortisol during the day. Situations of physical or psychological stress, such as infections, trauma or surgery, can result in a physiological increase in serum cortisol levels up to 150–200 mg [8, 9]. In some circumstances associated with intense inflammation, such as sepsis, the adrenal gland’s ability to produce cortisol may be insufficient to maintain vital function, a situation described as relative adrenal insufficiency [10].

The critical functions of glucocorticoids for survival involve the presence of receptors for these molecules in the regulation of gene transcription processes. The glucocorticoid receptor can function at least at three levels: first–recruitment of the general transcription machinery; second–modulation of transcription factor action through direct protein–protein interactions; and third–modulation of chromatin structure to allow the assembly of other gene regulatory proteins and the general transcription machinery on the DNA [11]. Interactions between corticosteroid receptors and target genes, especially the ones related to the immune system, seem to be among the mechanisms responsible for the anti-inflammatory effects observed with pharmacological doses of these medications [12].

In addition to the genomic effects, glucocorticoid have non-genomic actions of significant therapeutic relevance. From what is known so far, these effects are mediated by three different mechanisms: physicochemical interactions with cellular membranes, membrane-bound glucocorticoid receptor-mediated effects and cytosolic glucocorticoid receptor-mediated effects [13]. Although the mechanisms are not yet fully understood, the non-genomic actions of glucocorticoids may play a role in the management of inflammatory diseases [14].

Since 1950, when cortisol was first synthesized, several studies have been carried out with the aim of synthesizing a steroid with a specific anti-inflammatory action. It quickly became clear that it was possible to alter the steroid molecule so that the glucocorticoid or mineralocorticoid activity was intensified. However, the increase in anti-inflammatory activity is intrinsically associated with the increase in other glucocorticoid actions, which determines a significant metabolic effect [15]. Dexamethasone belongs to this group: higher glucocorticoid potency and minimal mineralocorticoid activity, generating greater hypothalamic–pituitary–adrenal axis suppression and more metabolic side effects than other corticosteroids.

### Metabolic effects of glucocorticoids

**Fig. 1:**
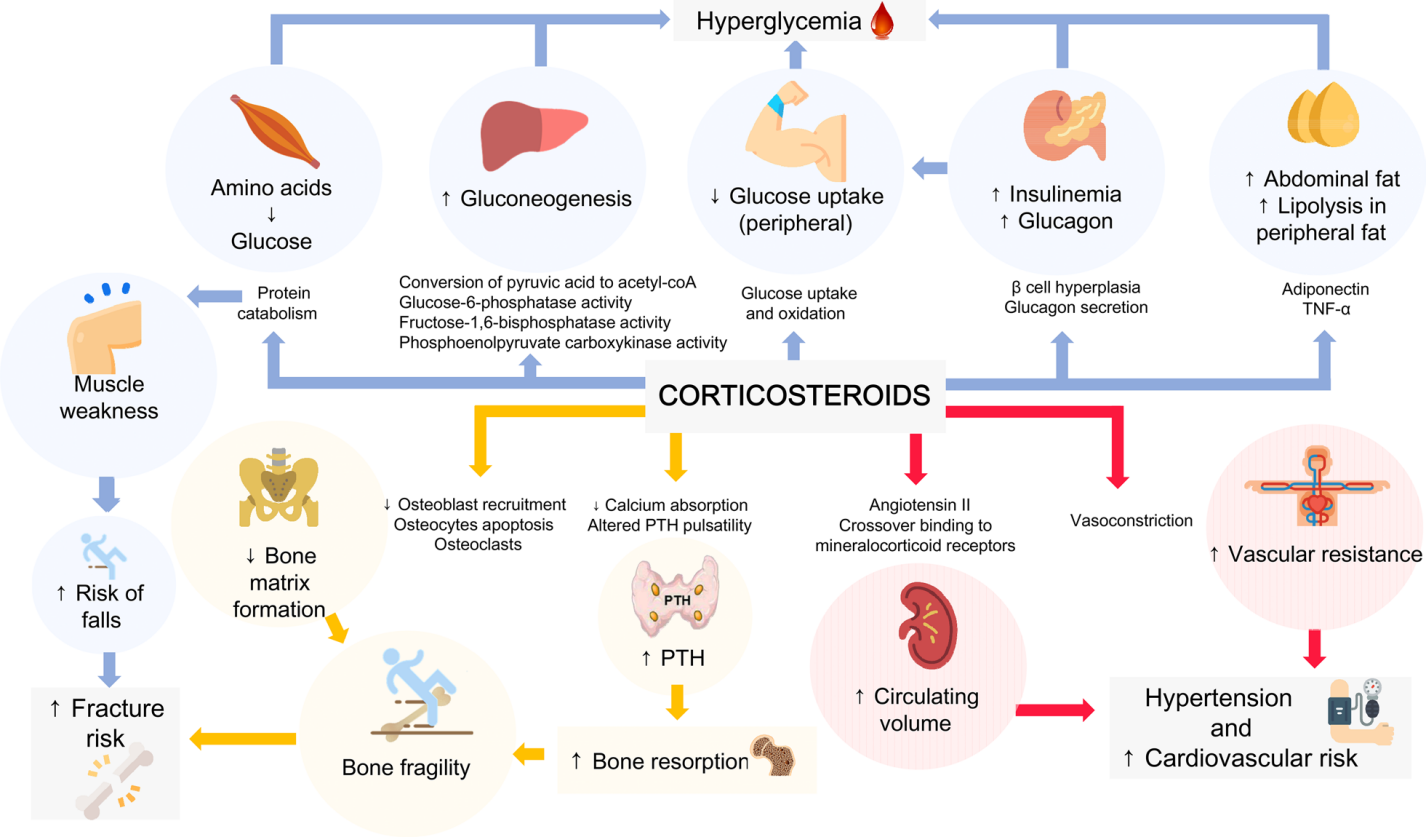
Effect of the use of corticosteroids on different functions of the body

The administration of glucocorticoids causes a significant change on the metabolism of carbohydrates, which can lead to insulin resistance, hyperglycemia and glycosuria. One of the first well-elucidated effects of this drug was its role in increasing hepatic gluconeogenesis, which seems to be related to the inhibitory effects of glucocorticoids on the conversion of pyruvic acid to acetyl-coenzyme A, leading to an accumulation of pyruvic acid and resulting in glucose resynthesis [15–17]. Increased induction of enzymes related to gluconeogenesis, such as glucose-6-phosphatase, fructose-1,6-bisphosphatase and phosphoenolpyruvate carboxykinase contribute to this effect [18]. An increase in hepatic glycogen deposition can be observed from three to twenty-four hours after the administration of glucocorticoids [19]. In addition, the use of glucocorticoids plays an important role by augmenting glucose production and decreasing peripheral glucose utilization, maintaining high serum glucose levels [20]. This action, which in physiological situations is fundamental for maintaining euglycemia during periods of fasting, may be exacerbated with the administration of exogenous corticosteroids, leading to hyperglycemia (see Fig. 1).

The chronic use of corticosteroids also increases fasting insulin levels [21]. This effect is related to a metabolic response of the pancreatic beta cells to hyperglycemia, which results in reduced peripheral sensitivity to insulin [22, 23]. In addition, glucocorticoids also alter insulin secretion by reducing the effect of incretins, even though this action has not yet been fully understood [21, 24, 25].

Experimental studies have shown that glucocorticoids have a pro-adipogenic function. The lipogenic effect of these drugs seems to be mediated by the genetic expression of pathways that lead to the maximization of insulin effects [35]. When evaluating animal models after exposure to the use of corticosteroids, high levels of these drugs in adipose tissue were associated with increased deposition of abdominal fat, reduced glucose tolerance and hypertriglyceridemia. Moreover, there was a reduction in adiponectin levels and an increase in serum tumor necrosis factor alpha (TNF-α) levels, which are related to insulin sensitivity and resistance, respectively [26–30]. Glucocorticoids also have a lipolytic action, especially pronounced in peripheral fat. This function is mediated by the induction of transcription factors that regulate the function of lipases, increasing the action of these enzymes [27, 31–34]. However, the acute and long-term effects of corticosteroids on lipolysis are still not entirely clear.

Glucocorticoids play a role in controlling liver metabolism mediated by the genomic regulation of glucocorticoids receptors. More than 50 target genes for this regulation have been identified so far [27, 36]. These genes codify enzymes responsible for lipogenesis and triglyceride synthesis, and the consequences of the activation of these enzymes can lead to the development of hepatic steatosis even before the establishment of insulin resistance in the metabolic syndrome [27, 37–40]. In relation to other lipoproteins, in vitro and in vivo studies have demonstrated that animals treated with dexamethasone showed an increase in serum very-low density lipoproteins (VLDL) and high-density lipoproteins (HDL). This effect is believed to be due to increased production of apolipoprotein B (ApoB), associated with increased synthesis of triglycerides and apolipoprotein A-I (ApoA1) respectively, which are stimulated by the use of corticosteroids [41–45]. In the case of low-density lipoproteins (LDL), the analyses so far are not conclusive, and further studies are needed to determine what the effect would be on these molecules [27].

Protein metabolism is also significantly affected by corticosteroids, which have shown to stimulate catabolism, resulting in inhibition of growth, osteoporosis, muscular atrophy, reduction in skin thickness and reduction in the amount of lymphoid tissue. Protein catabolism is the process by which proteins are broken down to their amino acids. Consequently, a greater uptake of amino acids in the liver occurs. These amino acids will be predominantly deaminated and converted to glucose or, less frequently, transformed into new proteins [15, 26].

Regarding calcium metabolism, glucocorticoids have a direct and indirect effect on bone remodeling [46]: they inhibit the formation of the bone matrix, which seems to be related to the reduction of the osteoblast’s recruitment and to the accelerated apoptosis of osteocytes [47]. Another effect that can be observed is the increase in the expression of receptor activator of nuclear factor-κB ligand (RANKL), which leads to increases in the number of bone-resorbing osteoclasts. This condition may be accompanied by other changes, such as reduced muscle mass, which can be present due to protein catabolism, and with cataract-related visual impairment, which is more prevalent in corticosteroid users. These three factors combined result in a significant increase in the risk of falls and fractures even before observation of bone mineral density reduction. Other indirect effects that can be observed are the reduction in calcium reabsorption in the kidney, changes in sex hormones and changes in the parathyroid hormone pulsatility, factors that are fundamental for adequate bone homeostasis [46, 48].

Water and sodium retention and the reduction in serum potassium are complications of the use of corticosteroids, especially those with mineralocorticoid action and when high doses are administered [15]. There are currently several formulations available with a predominance of glucocorticoid effect and practically negligible mineralocorticoid effect, with dexamethasone as an example of this category. Despite this, the effect on water regulation remains, regardless of the mineralocorticoid effect. Glucocorticoids act indirectly in the proximal tubule, increasing the cellular response of sodium transporters stimulated by angiotensin II. In the distal tubule, the effect is more direct and seems to be related to crossover binding to mineralocorticoid receptors. As a result, there is an increase in sodium and water retention, increasing the circulating volume and causing an increase in blood pressure levels [48, 50]. Another indirect effect of glucocorticoids that results in arterial hypertension is the magnification of the circulating vasoconstrictors’ response, since it acts upregulating the expression of receptors to many vasoconstrictors and downregulating the effects of potential vasodilators [51–55]. Thus, glucocorticoids have the potential to alter both circulating volume and vascular resistance [49].

### Corticosteroids and COVID-19

Since December 2019, when a series of cases of severe pneumonia caused by the SARS-CoV-2 coronavirus were described in China, several studies have sought to evaluate the effect of corticosteroids on the natural course of the disease [56–58]. The rationale for the use of dexamethasone in patients with severe infection is based on this premise that the damage caused by the disease is strongly related to the aggressive inflammatory response triggered [59]. Thus, the use of drugs with a potent anti-inflammatory effect could reduce the catastrophic effects generated by the overactivation of the immune system, helping to speed up the recovery of these patients [60]. The results published so far are, however, contradictory and inconclusive.

A study in vitro by Matsuyama et al. investigated the effect of inhaled corticosteroids on the replication of the SARS-CoV. This study suggested that ciclesonide interacts with the newly mapped coronavirus protein NSP15 during biogenesis and suppresses viral replication of SARS-CoV-2. Inhaled ciclesonide is expected to reduce viral replication and host inflammation in the lungs, with decreased immunosuppressive effects compared to systemic corticosteroids, as ciclesonide primarily remains in the lung tissue [61]. Later, a series of case reports by Iwabuchi et al. using this medication was published, showing favorable results [62].

Another study conducted by Wang et al. included 46 hospitalized patients with severe COVID-19 pneumonia, who were divided into two groups based on whether they received or not corticosteroid treatment. The first group received methylprednisolone (1–2 mg/kg/d for 5–7 days), and the second group received standard therapy without methylprednisolone. The first group achieved faster improvement in clinical symptoms (fever and peripheral oxygen saturation) and lung lesions detected by imaging. However, there was no improvement in mortality or in laboratory parameters [63].

A meta-analysis carried out by Lee et al., published in April 2020, evaluated studies from January 2002 to March 2020 that included patients with severe coronavirus pneumonia, and found an increase in mortality, duration of hospitalization and rates of associated secondary bacterial infection in patients treated with corticosteroids [64]. Other meta-analyses carried out later showed similar results [65, 66]. Despite that, all meta-analyses included studies of other coronavirus outbreaks, and there was still a lack of robust and quality work that specifically assessed the impact of these medications on serious SARS-CoV-2 infections.

The RECOVERY (Randomised Evaluation of COVID-19 Therapy) study, first large clinical trial conducted to assess the impact of the use of dexamethasone on COVID-19 infection, was published in July 2020. This study analysed 2104 patients who received 6 mg dexamethasone daily compared to participants who received usual care. A reduction in mortality of up to one-third (29.3% vs. 41.4%; rate ratio, 0.64; 95% CI 0.51 to 0.81) in patients on mechanical ventilation and up to one-fifth (23.3% vs. 26.2%; rate ratio, 0.82; 95% CI 0.72 to 0.94) in patients receiving only oxygen was found. There were no benefits in patients without the need for ventilatory support [6]. Although it was recently published, the results already have great repercussion in the media and social networks. The impact it will have on the general population is still unknown.

The results of the RECOVERY trial must be looked at carefully. It is important to notice that the patients that benefited most from the use of dexamethasone, which were the ones receiving invasive mechanical ventilation, were on average 10 years younger than those not receiving any respiratory support and had a history of a longer duration of symptoms (an average of 7 days longer) [6]. Also, despite the promising results related to the reduction of mortality in cases of severe COVID-19 infection, this study has methodological limitations that should be critically considered. Changes in inclusion criteria, such as expanding the age limit during the course of the study, were not anticipated in the clinical trials’ protocol. The withdrawal of patients from the trial after the randomization in case the dexamethasone was unavailable generates a margin for bias. In addition, the short follow-up time (28 days) makes interpretation difficult in relation to hospitalized and severely ill patients who still had an uncertain prognosis at the end of this period. In relation to patients with diabetes, this population appears to have been adequately sampled in the study, with a co-prevalence of diabetes and COVID-19 similar to that described in the literature. It is an important limitation of the study not to describe the occurrence of hyperglycemia at different times during and after the use of dexamethasone, considering the potential risks of this complication in patients with diabetes.

It is necessary to remember that the use of corticosteroids in critically ill patients is still controversial. Several systematic reviews carried out on the topic have shown contradictory results on the benefits of hydrocortisone in mortality in shock situations [67–70]. Taking this into account, the Surviving Sepsis Campaign 3 guideline suggests against the use of corticosteroids to treat septic shock in patients with adequate resuscitation with fluids and vasopressors. The use of hydrocortisone at a dose of 200 mg per day would be indicated only for refractory cases, but with a weak recommendation [71]. The controversial results also apply to the use of corticosteroids in patients with acute respiratory distress syndrome, on which different meta-analyses carried out in recent years have presented divergent results, making this difficult to value for clinical practice [72–75]. So far, the use of corticosteroids in critically ill patients, regardless of etiology, remains restricted to cases in which there is refractoriness in shock or in which the ventilatory pattern reflects bronchial hyperreactivity [76]. There is still a long way to go before the validation of the presented results and the incorporation of these medications in the care of the patient infected with the coronavirus, if appropriate.

The possible inadvertent consumption of dexamethasone after the release of the results of the RECOVERY study could be catastrophic. The effects of mass dissemination of these results will soon be apparent to our population. Fear and uncertainty in the face of a potentially serious infection may lead lay people to desperate decisions when looking for a “magic pill”. This feeling may lead to self-medication and the inappropriate and abusive use of corticosteroids, similarly to what happened after the disclosure of other drugs. This concern is especially relevant in countries where some corticosteroids can be dispensed without a medical prescription, such as Brazil.

### Corticosteroids and diabetes

Chronic or high-dose use of corticosteroids can, by itself, lead to the onset of diabetes, especially in previously insulin-resistant or obese individuals. Changes in carbohydrate metabolism, including insulin resistance and reduced peripheral glucose uptake related to its use, may explain the increased risk of diabetes. Epidemiology data on predisposing factors and the prevalence of corticosteroid-induced diabetes are not well known; however, it is estimated to occur in up to 20 to 54% of people treated with corticosteroids [77, 78]. It is postulated that patients who have a predisposition, such as the presence of Langerhans B cells with latent dysregulation or some alteration in previous peripheral sensitivity, are at greater risk of developing diabetes. Some of the risk factors for corticosteroid-induced diabetes are the same as those for type 2 diabetes: age, family history of diabetes, previous gestational diabetes, and abdominal obesity. It is known that the drug use duration and the cumulative dose used are direct predictors of higher risk for diabetes [79].

Even more damaging is the effect of glucocorticoids on patients who already have the diagnosis of diabetes. Studies show that hyperglycemia and insulin resistance enhanced by the use of glucocorticoids results in uncontrolled diabetes in a dose-dependent manner [80, 81]. In addition to all the complications already known of inappropriate glycemic control, especially regarding the presence of micro and macrovascular complications, another study also demonstrated that elderly patients with diabetes on chronic use of high daily doses of corticosteroids had a 94% higher risk of being hospitalized due to diabetes complications [82].

In addition to the direct effects on glycemic control, the other previously mentioned effects on arterial pressure control, lipid and bone metabolism also have a potential for severe consequences in patients with diabetes. Hypertension, obesity and diabetes are diseases that are intrinsically connected in patients with metabolic syndrome. The vasoconstrictor effects and the increase in circulating volume, resulting in uncontrolled blood pressure, as well as the redistribution of fat, resulting in increased incidence of hepatic steatosis, can be especially harmful in this group. In addition, changes in bone mineral and muscle mass in patients who already have bone fragility related to diabetes can result in an increased risk of falls and fractures [83, 84]. Lastly, the use of high doses or the long-term use (more than seven days) of corticosteroids would lead to hypothalamic–pituitary–adrenal axis suppression. Drug withdrawal should be carefully done in order not to induce an iatrogenic adrenal crisis.

In patients with either a steroid-induced diabetes or a previous diagnosis of diabetes using corticosteroids, it will be required to pay close attention to blood glucose monitoring, and an early intervention may be necessary to prevent prolonged symptomatic hyperglycemia. There is no consensus on which glycemic targets are ideal for patients using glucocorticoids. In patients without a previous diagnosis of diabetes but who are at high risk of hyperglycemia (family history of diabetes, previous gestational diabetes, pre-diabetes, polycystic ovary syndrome, obesity), we suggest considering assessment of glycemic control daily for those who use doses greater than the equivalent of 40 mg of prednisone daily for periods greater than 7–14 days, although one guideline suggests a more frequent glycemic verification [85]. In case of blood glucose levels greater than 180 mg/dL, consider a more frequent observation routine, which should be individualized for each case. For patients with a previous diagnosis of diabetes, it is suggested to reinforce the testing routine before meals during the treatment with corticosteroids [85]. We recommend, according to Suh et al., that the treatment for hyperglycemia be discussed and possibly considered when the preprandial and postprandial capillary glucose levels are ≥ 140 and ≥ 200 mg/dL, respectively [86]. Treatment adjustments should be made according to guidelines for the treatment of diabetes.

There is no treatment strategy that is ideal for all patients, and the choice of the regimen to be used will depend on which corticosteroid is in use, its potency and the duration of its action. For short-acting corticosteroids, such as prednisone, the plasma peak occurs in four to six hours after administration, but the pharmacological actions can last throughout the day [85]. It is common for patients who use only morning doses to experience glycemic oscillations throughout the day, with a tendency to normalize blood glucose levels at night [85]. For long-acting corticosteroids, such as dexamethasone, or for regimens using multiple doses, glycemic changes can last longer, affecting also fasting glucose [87, 88].

The American Diabetes Association recommends the use of insulin to correct glycemic oscillations in patients using corticosteroids. For patients receiving only single morning doses of short-acting corticosteroids, the use of intermediate or long-acting insulin analogues is usually the standard approach. For long-acting glucocorticoids, multidose or continuous glucocorticoid use, long-acting insulin may be required to control fasting blood glucose. For higher doses of glucocorticoids, a basal bolus insulin approach is often needed [89]. One recommendation is to initiate or to adjust weight-based NPH insulin at 0.1 units/kg for every 10 mg of prednisone up to a maximum of 0.4 units/kg initially [90]. Other treatment options, especially for treatment naive patients, include using sulfonylureas and thiazolidinediones considering their effects on prandial blood glucose and improvement in insulin sensitivity via PPAR agonism, respectively. It is necessary to keep in mind the risks of these medications, considering that sulfonylureas carry a risk of hypoglycemia, while thiazolidinediones are associated with fluid retention [91]. Evidences for glucagon-like peptide-1 receptor agonists, dipeptidyl peptidase-4 inhibitors or sodium-glucose co-transporter 2 inhibitors in corticosteroids use are still limited.

Planning to withdraw corticosteroids treatment should also be part of the approach in cases where there is a prolonged use of these drugs. The most feared complication in corticosteroid withdrawal is the suppression of the hypothalamic–pituitary–adrenal axis, which can result in secondary adrenal insufficiency [92, 93]. Symptoms of chronic adrenal insufficiency include abdominal pain, nausea and vomiting, postural hypotension, drowsiness, anorexia, weakness, myalgia, arthralgia and depression [93]. More severe cases can manifest with vomiting, diarrhea, fever, acute dehydration, hypotension, shock and coma, characterizing an acute adrenal insufficiency, which is a life-threatening situation [94]. Studies show that patients who use corticosteroids for more than 14 days are at risk of developing adrenal insufficiency and need to gradually reduce the dose of the medication [95, 96]. Considering that COVID-19 is a self-limiting infection disease, it is possible that the use of corticosteroids occurs for a shorter period of time, without causing any major concerns regarding the suppression of the hypothalamic–pituitary–adrenal axis. However, for patients who require prolonged use, gradual withdrawal strategies should be considered.

There are several different protocols for the gradual reduction of the corticosteroids; however, only a few have been tested in clinical trials, with no conclusive results. Currently, there is no evidence to support the use of one over the other. Some characteristics are associated with a lower chance of developing adrenal insufficiency in corticosteroid removal. The use of the total dose in the morning, avoiding night doses, may be a good strategy, considering that the night dose can block the peak of morning ACTH, increasing the chance of blocking the hypothalamic–pituitary–adrenal axis [97]. The administration of the total dose on alternate days is another strategy, and the rationale for its use is based on the theory that the anti-inflammatory effect persists longer than the undesired metabolic effects, making this an alternative for a lower incidence of adverse effects.

**Fig. 2:**
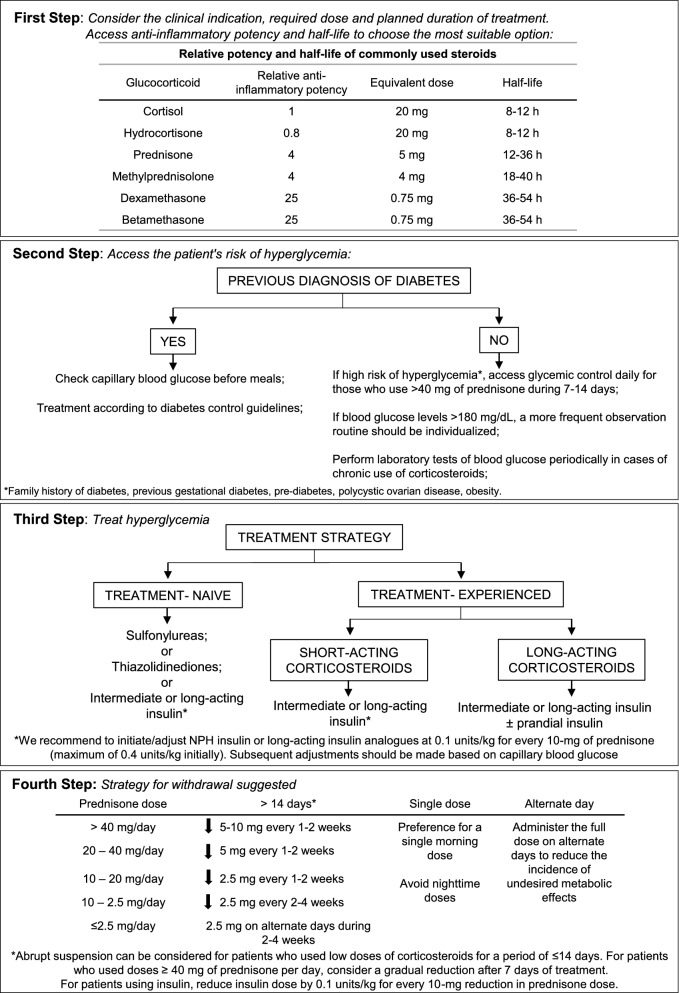
Rational assessment for the initiation, maintenance and withdrawal of corticosteroids

According to Axelrod et al., for patients who used corticosteroids for a period of less than or equal to 7 days, medication withdrawal can be done abruptly, without risk of adrenal suppression [95]. More recent references suggest that, when used for less than 14 days, there would be a low risk of hypothalamic–pituitary–adrenal axis suppression [96]. We suggest that the time for abrupt suspension should be assessed individually, and can be considered for patients who used low doses of corticosteroids for a period of less than or equal to 14 days. For those who used higher doses (greater than or equal to the equivalent of 40 mg of prednisone per day) for more than 7 days, we suggest a gradual dose reduction. When treatment is carried out for a longer period than 14 days, a gradual reduction according to the total dose administered is usually recommended. We suggest, for patients using a daily dose greater than 40 mg of prednisone (or equivalent), to start with a reduction of 5 to 10 mg every 1 or 2 weeks. When the daily dose is between 20 and 40 mg of prednisone (or equivalent), we suggest a reduction of 5 mg every 1 or 2 weeks. When the daily dose is between 10 and 20 mg of prednisone (or equivalent), we suggest a reduction of 2.5 mg every 1 or 2 weeks [98]. When the daily dose is less than 10 mg of prednisone (or equivalent), we suggest reducing 2.5 mg every 2 to 4 weeks and then administer 2.5 mg of prednisone on alternate days during 2 to 4 weeks until complete suspension. It is important to remember that, for patients using insulin, dose adjustment should be performed during the corticosteroid removal. Wallace et al. recommends reducing insulin dose by 0.1 units/kg for every 10-mg reduction in the prednisone dose [91]. A summary of the suggested approach is shown in Fig. 2.

## Conclusion

The presence of hyperglycemia, increased insulin resistance and increased blood pressure levels are some of the effects that may clinically decompensate the patient with diabetes who decides to use corticosteroids without medical supervision or those being prescribed by physicians not aware of how to handle this situation. In the long run, the effects on liver metabolism, leading to hypertriglyceridemia and steatosis, and on bone metabolism, leading to a reduction in bone matrix, may lead to even more serious consequences. Although it is well known that these effects are dose dependent, it is unknown how the use of this medication, without medical supervision, will occur. However, it may occur in an erratic or lasting way. The risks of adrenal insufficiency after abrupt withdrawal of dexamethasone or Cushing’s syndrome with continuous and prolonged use should always be suspected. More than ever, it is necessary for health professionals to be alert and able to predict damages related to the use of these drugs, which is the first step to minimize the potential damages to come. It is essential that, at this time, patients be offered guidance on the danger of inappropriate use of corticosteroids and self-prescription without adequate medical monitoring. Inexperience and recklessness in the use of medications such as dexamethasone have the power to transform a friendly drug into a potential foe of our health.

## Data Availability

Since it is a narrative review, availability of data and materials does not apply.

## References

[1] Gautret P, Lagier JC, Parola P, Hoang VT, Meddeb L, Mailhe M (2020). Hydroxychloroquine and azithromycin as a treatment of COVID-19: results of an open-label non-randomized clinical trial. Int J Antimicrob Agents.

[2] Oliva G. Coronavírus: desaparecimento da cloroquina das farmácias do Rio preocupa pacientes usuários de medicamentos. O Globo, Rio de Janeiro, 21 de março de 2020. Sociedade. https://oglobo.globo.com/sociedade/coronavirus-servico/coronavirus-desaparecimento-da-cloroquina-das-farmacias-do-rio-preocupa-pacientes-usuarios-de-medicamentos-24318959. Acessed 20 2020.

[3] Verdélio A. Brasil recebe 2 milhões de doses de hidroxicloroquina dos EUA. Agência Brasil, Brasília, 01 de junho de 2020. https://agenciabrasil.ebc.com.br/politica/noticia/2020-06/brasil-recebe-dois-milhoes-de-doses-de-hidroxicloroquina-dos-eua. Acessed 20 2020.

[4] Geleris J, Sun Y, Platt J, Zucker J, Baldwin M, Hripcsak G (2020). Observational Study of Hydroxychloroquine in Hospitalized Patients with Covid-19. N Engl J Med.

[5] Tang W, Cao Z, Han M, Wang Z, Chen J, Sun W (2020). Hydroxychloroquine in patients with mainly mild to moderate coronavirus disease 2019: open label, randomised controlled trial. BMJ.

[6] Horby P, Lim WS, RECOVERY Collaborative Group (2020). Dexamethasone in Hospitalized patients with Covid-19–Preliminary Report. N Engl J Med.

[7] Moss GP (1989). Nomenclature of steroids. Pure Appl Chem.

[8] Gupta P, Bhatia V (2008). Corticosteroid physiology and principles of therapy. Indian J Pediatr.

[9] Williams DM (2018). Clinical Pharmacology of Corticosteroids. Respir Care.

[10] Annane D (2008). Adrenal insufficiency in sepsis. Curr Pharm Des.

[11] McEwan IJ, Wright AP, Gustafsson JA (1997). Mechanism of gene expression by the glucocorticoid receptor: role of protein-protein interactions. BioEssays.

[12] Coutinho AE, Chapman KE (2011). The anti-inflammatory and immunosuppressive effects of glucocorticoids, recent developments and mechanistic insights. Mol Cell Endocrinol.

[13] Song IH, Buttgereit F (2006). Non-genomic glucocorticoid effects to provide the basis for new drug developments. Mol Cell Endocrinol.

[14] Panettieri RA, Schaafsma D, Amrani Y, Koziol-White C, Ostrom R, Tliba O (2019). Non-genomic effects of glucocorticoids: an updated view. Trends Pharmacol Sci.

[15] Binder C (1969). The physiology and pharmacology of the glucocorticoids. Acta Med Scand Suppl.

[16] de Bodo RC, Altszuler N (1958). Insulin hypersensitivity and physiological insulin antagonists. Physiol Rev.

[17] Thorn GW, Renold AE, Winegrad AI (1957). Some effects of adrenal cortical steroids on intermediary metabolism. Br Med J.

[18] Cassuto H, Kochan K, Chakravarty K, Cohen H, Blum B, Olswang Y (2005). Glucocorticoids regulate transcription of the gene for phosphoenolpyruvate carboxykinase in the liver via an extended glucocorticoid regulatory unit. J Biol Chem.

[19] Ashmore J, Morgan D. Metabolic effects of adrenal glucocorticoid hormones. Eisenstein, A.B: The adrenal cortex, London, 1967. (1)249-267.

[20] De Feo P, Perriello G, Torlone E, Ventura MM, Fanelli C, Santeusanio F (1989). Contribution of cortisol to glucose counterregulation in humans. Am J Physiol.

[21] Fichna M, Fichna P (2017). Glucocorticoids and beta-cell function. Endokrynol Pol.

[22] Lenzen S, Bailey CJ (1984). Thyroid hormones, gonadal and adrenocortical steroids and the function of the islets of Langerhans. Endocr Rev.

[23] Karlsson S, Ostlund B, Myrsén-Axcrona U, Sundler F, Ahrén B (2001). Beta cell adaptation to dexamethasone-induced insulin resistance in rats involves increased glucose responsiveness but not glucose effectiveness. Pancreas.

[24] Richter G, Göke R, Göke B, Arnold R (1990). Dexamethasone pretreatment of rat insulinoma cells decreases binding of glucagon-like peptide-1(7-36)amide. J Endocrinol.

[25] Sato T, Hayashi H, Hiratsuka M, Hirasawa N (2015). Glucocorticoids decrease the production of glucagon-like peptide-1 at the transcriptional level in intestinal L-cells. Mol Cell Endocrinol.

[26] Knox WE, Auerbach VH, Lin EC (1956). Enzymatic and metabolic adaptations in animals. Physiol Rev.

[27] de Guia RM, Herzig S (2015). How Do Glucocorticoids Regulate Lipid Metabolism?. Adv Exp Med Biol.

[28] Masuzaki H, Paterson J, Shinyama H, Morton NM, Mullins JJ, Seckl JR (2001). A transgenic model of visceral obesity and the metabolic syndrome. Science.

[29] Viengchareun S, Zennaro MC, Pascual-Le Tallec L, Lombes M (2002). Brown adipocytes are novel sites of expression and regulation of adiponectin and resistin. FEBS Lett.

[30] Fasshauer M, Klein J, Neumann S, Eszlinger M, Paschke R (2002). Hormonal regulation of adiponectin gene expression in 3T3-L1 adipocytes. Biochem Biophys Res Commun.

[31] Peckett AJ, Wright DC, Riddell MC (2011). The effects of glucocorticoids on adipose tissue lipid metabolism. Metabolism.

[32] Yu CY, Mayba O, Lee JV, Tran J, Harris C, Speed TP (2010). Genome-wide analysis of glucocorticoid receptor binding regions in adipocytes reveal gene network involved in triglyceride homeostasis. PLoS ONE.

[33] Campbell JE, Peckett AJ, Dsouza AM, Hawke TJ, Riddell MC (2011). Adipogenic and lipolytic effects of chronic glucocorticoid exposure. Am J Physiol Cell Physiol.

[34] Ebbert JO, Jensen MD (2013). Fat depots, free fatty acids, and dyslipidemia. Nutrients.

[35] Hillgartner FB, Salati LM, Goodridge AG (1995). Physiological and molecular mechanisms involved in nutritional regulation of fatty acid synthesis. Physiol Rev.

[36] van den Berghe G (1991). The role of the liver in metabolic homeostasis: implications for inborn errors of metabolism. J Inherit Metab Dis.

[37] Legrand P, Catheline D, Hannetel JM, Lemarchal P (1994). Stearoyl-CoA desaturase activity in primary culture of chicken hepatocytes. Influence of insulin, glucocorticoid, fatty acids and cordycepin. Int J Biochem.

[38] Dich J, Bro B, Grunnet N, Jensen F, Kondrup J (1983). Accumulation of triacylglycerol in cultured rat hepatocytes is increased by ethanol and by insulin and dexamethasone. Biochem J.

[39] Dolinsky VW, Douglas DN, Lehner R, Vance DE (2004). Regulation of the enzymes of hepatic microsomal triacylglycerol lipolysis and re-esterification by the glucocorticoid dexamethasone. Biochem J.

[40] Mangiapane EH, Brindley DN (1986). Effects of dexamethasone and insulin on the synthesis of triacylglycerols and phosphatidylcholine and the secretion of very-low-density lipoproteins and lysophosphatidylcholine by monolayer cultures of rat hepatocytes. Biochem J.

[41] aylor AH, Raymond J, Dionne JM, Romney J, Chan J, Lawless DE, et al. Glucocorticoid increases rat apolipoprotein A-I promoter activity. J Lipid Res. 1996;37(10):2232-43. 8906599

[42] Martin-Sanz P, Vance JE, Brindley DN (1990). Stimulation of apolipoprotein secretion in very-low-density and high-density lipoproteins from cultured rat hepatocytes by dexamethasone. Biochem J.

[43] Duerden JM, Bartlett SM, Gibbons GF (1989). Long-term maintenance of high rates of very-low-density-lipoprotein secretion in hepatocyte cultures. A model for studying the direct effects of insulin and insulin deficiency in vitro. Biochem J.

[44] Wang CN, Hobman TC, Brindley DN (1995). Degradation of apolipoprotein B in cultured rat hepatocytes occurs in a post-endoplasmic reticulum compartment. J Biol Chem.

[45] Hazra A, Pyszczynski NA, DuBois DC, Almon RR, Jusko WJ (2008). Modeling of corticosteroid effects on hepatic low-density lipoprotein receptors and plasma lipid dynamics in rats. Pharm Res.

[46] Buckley L, Humphrey MB (2018). Glucocorticoid-Induced Osteoporosis. N Engl J Med.

[47] Panday K, Gona A, Humphrey MB (2014). Medication-induced osteoporosis: screening and treatment strategies. Ther Adv Musculoskelet Dis.

[48] Weinstein RS (2012). Glucocorticoid-induced osteoporosis and osteonecrosis. Endocrinol Metab Clin North Am.

[49] Brem AS (2001). Insights into glucocorticoid-associated hypertension. Am J Kidney Dis.

[50] Kenouch S, Alfaidy N, Bonvalet JP, Farman N (1994). Expression of 11 beta-OHSD along the nephron of mammals and humans. Steroids.

[51] Ullian ME, Walsh LG (1995). Corticosterone metabolism and effects on angiotensin II receptors in vascular smooth muscle. Circ Res.

[52] Brem AS, Bina RB, Hill N, Alia C, Morris DJ (1997). Effects of licorice derivatives on vascular smooth muscle function. Life Sci.

[53] Brem AS, Bina RB, King T, Morris DJ (1997). 11BetaOH-progesterone affects vascular glucocorticoid metabolism and contractile response. Hypertension.

[54] Hackenthal E, Klett C (1993). Angiotensin II and dexamethasone regulate angiotensinogen mRNA by different mechanisms. J Steroid Biochem Mol Biol.

[55] Fishel RS, Eisenberg S, Shai SY, Redden RA, Bernstein KE, Berk BC (1995). Glucocorticoids induce angiotensin-converting enzyme expression in vascular smooth muscle. Hypertension.

[56] Zhu N, Zhang D, Wang W, Li X, Yang B, Song J (2020). A novel coronavirus from patients with pneumonia in China, 2019. N Engl J Med.

[57] Gorbalenya AE, Baker SC, Baric RS, et al. Severe acute respiratory syndrome-related coronavirus: The species and its viruses–a statement of the Coronavirus Study Group. BioRxiv. 2020: 1-15.

[58] Li Q, Guan X, Wu P, Wang X, Zhou L, Tong Y (2020). Early transmission dynamics in Wuhan, China, of Novel coronavirus-infected pneumonia. N Engl J Med.

[59] Huang C, Wang Y, Li X, Ren L, Zhao J, Hu Y (2020). Clinical features of patients infected with 2019 novel coronavirus in Wuhan, China. Lancet.

[60] Saghazadeh A, Rezaei N (2020). Towards treatment planning of COVID-19: rationale and hypothesis for the use of multiple immunosuppressive agents: Anti-antibodies, immunoglobulins, and corticosteroids. Int Immunopharmacol.

[61] Matsuyama S, Kawase M, Nao N, Shirato K, Ujke M, Kamitani W, et al. The inhaled corticosteroid ciclesonide blocks coronavirus RNA replication by targeting viral NSP 15. Microbiology. 2020; internet. http://biorxiv.org/lookup/doi/10.1101/2020.03.11.987016. Acessed 20 2020.

[62] Iwabuchi K, Yoshie K, Kurakami Y, Takahashi K, Kato Y, Morishima T (2020). Therapeutic potential of ciclesonide inahalation for COVID-19 pneumonia: report of three cases. J Infect Chemother.

[63] Wang Y, Jiang W, He Q, Wang C, Wang B, Zhou P, et al. Early, low-dose and short-term application of corticosteroid treatment in patients with severe COVID-19 pneumonia: single-center experience from Wuhan, China. Infectious Diseases; 2020. http://medrxiv.org/lookup/doi/10.1101/2020.03.06.20032342. Accessed 20 Jun 2020.

[64] Yang Z, Liu J, Zhou Y, Zhao X, Zhao Q (2020). The effect of corticosteroid treatment on patients with coronavirus infection: a systematic review and meta-analysis. J Infect.

[65] Li H, Chen C, Hu F, Wang J, Zhao Q, Gale RP (2020). Impact of corticosteroid therapy on outcomes of persons with SARS-CoV-2, SARS-CoV, or MERS-CoV infection: a systematic review and meta-analysis. Leukemia.

[66] Ye Z, Wang Y, Colunga-Lozano LE, Prasad M, Tangamornsuksan W, Rochwerg B, et al. Efficacy and safety of corticosteroids in COVID-19 based on evidence for COVID-19, other coronavirus infections, influenza, community-acquired pneumonia and acute respiratory distress syndrome: a systematic review and meta-analysis. CMAJ. 2020: 1-12. 10.1503/cmaj.200645 PMC7828900 32409522

[67] Annane D, Bellissant E, Bollaert PE, Briegel J, Confalonieri M, De Gaudio R (2009). Corticosteroids in the treatment of severe sepsis and septic shock in adults: a systematic review. JAMA.

[68] Sligl WI, Milner DA, Sundar S, Mphatswe W, Majumdar SR (2009). Safety and efficacy of corticosteroids for the treatment of septic shock: a systematic review and meta-analysis. Clin Infect Dis.

[69] Annane D, Bellissant E, Bollaert PE, Briegel J, Keh D, Kupfer Y. Corticosteroids for treating sepsis. Cochrane Database Syst Rev. 2015(12):CD002243. 10.1002/14651858.CD002243.pub3 PMC6494587 26633262

[70] Volbeda M, Wetterslev J, Gluud C, Zijlstra JG, van der Horst IC, Keus F (2015). Glucocorticosteroids for sepsis: systematic review with meta-analysis and trial sequential analysis. Intensive Care Med.

[71] Rhodes A, Evans LE, Alhazzani W, Levy MM, Antonelli M, Ferrer R (2017). Surviving sepsis campaign: International Guidelines for Management of Sepsis and Septic Shock: 2016. Crit Care Med.

[72] Horita N, Hashimoto S, Miyazawa N, Fujita H, Kojima R, Inoue M (2015). Impact of Corticosteroids on Mortality in Patients with Acute Respiratory Distress Syndrome: a Systematic Review and Meta-analysis. Intern Med.

[73] Zhao Q, Shi JX, Hu R, Li Q, Zhang CY, Li JS (2019). Effect of glucocorticoids on mortality in patients with acute respiratory distress syndrome: a meta-analysis. Exp Ther Med.

[74] Mammen MJ, Aryal K, Alhazzani W, Alexander PE (2020). Corticosteroids for patients with acute respiratory distress syndrome: a systematic review and meta-analysis of randomized trials. Pol Arch Intern Med.

[75] Zhou Y, Fu X, Liu X, Huang C, Tian G, Ding C (2020). Use of corticosteroids in influenza-associated acute respiratory distress syndrome and severe pneumonia: a systemic review and meta-analysis. Sci Rep.

[76] Lamontagne F, Quiroz Martinez H, Adhikari NK, Cook DJ, Koo KK, Lauzier F (2013). Corticosteroid use in the intensive care unit: a survey of intensivists. Can J Anaesth.

[77] De Micheli A. Corticosteroid induced diabetes mellitus: diagnosis and management. G Ital Nefrol. 2016;33(S68). 27960015

[78] Suissa S, Kezouh A, Ernst P (2010). Inhaled corticosteroids and the risks of diabetes onset and progression. Am J Med.

[79] Gagliardi L, Le Jeunne C (2012). Corticosteroids and diabetes mellitus. Presse Med.

[80] Slatore CG, Bryson CL, Au DH (2009). The association of inhaled corticosteroid use with serum glucose concentration in a large cohort. Am J Med.

[81] Blackburn D, Hux J, Mamdani M (2002). Quantification of the risk of corticosteroid-induced diabetes mellitus among the elderly. J Gen Intern Med.

[82] Caughey GE, Preiss AK, Vitry AI, Gilbert AL, Roughead EE (2013). Comorbid diabetes and COPD: impact of corticosteroid use on diabetes complications. Diab Care.

[83] Napoli N, Chandran M, Pierroz DD, Abrahamsen B, Schwartz AV, Ferrari SL (2017). Mechanisms of diabetes mellitus-induced bone fragility. Nat Rev Endocrinol.

[84] Sellmeyer DE, Civitelli R, Hofbauer LC, Khosla S, Lecka-Czernik B, Schwartz AV (2016). Skeletal metabolism, fracture risk, and fracture outcomes in type 1 and type 2 diabetes. Diabetes.

[85] Roberts A, James J, Dhatariya K (2018). Care JBDSJfI Management of hyperglycaemia and steroid (glucocorticoid) therapy: a guideline from the Joint British Diabetes Societies (JBDS) for Inpatient Care group. Diabet Med.

[86] Suh S, Park MK (2017). Glucocorticoid-induced diabetes mellitus: an important but overlooked problem. Endocrinol Metab.

[87] Kwon S, Hermayer KL, Hermayer K (2013). Glucocorticoid-induced hyperglycemia. Am J Med Sci.

[88] 89.Corsino L, Dhatariya K, Umpierrez G. Management of diabetes and hyperglycemia in hospitalized patients. In Endotext. http://www.ncbi.nlm.nih.gov/books/NBK279093/. Accessed 24 Jun 2020.

[89] Association AD (2020). Diabetes Care in the Hospital. Diabet Care.

[90] Clore JN, Thurby-Hay L (2009). Glucocorticoid-induced hyperglycemia. Endocr Pract.

[91] Wallace MD, Metzger NL (2018). Optimizing the Treatment of Steroid-Induced Hyperglycemia. Ann Pharmacother.

[92] Lamberts SW, Bruining HA, de Jong FH (1997). Corticosteroid therapy in severe illness. N Engl J Med.

[93] Dorin RI, Qualls CR, Crapo LM (2003). Diagnosis of adrenal insufficiency. Ann Intern Med.

[94] Shulman DI, Palmert MR, Kemp SF (2007). Adrenal insufficiency: still a cause of morbidity and death in childhood. Pediatrics.

[95] Axelrod L (1976). Glucocorticoid Therapy. Medicine.

[96] 97.Alexandraki KI, Kaltsas GA, Chrousos GP. Adrenal Suppression, 2018 Oct 1. In: Feingold KR, Anawalt B, Boyce A, et al., editors. Endotext [Internet]. South Dartmouth (MA): MDText.com, Inc.; 2000. https://www.ncbi.nlm.nih.gov/books/NBK279047/. Acessed 26 Jun 2020.

[97] Alves C, Robazzi TC, Mendonça M (2008). Withdrawal from glucocorticosteroid therapy: clinical practice recommendations. J Pediatr.

[98] De Oliveira EB. Paciente em uso prolongado de corticoide oral: quando/como fazer a retirada gradual? TelessaúdeRS, Porto Alegre, 05 de abril de 2017. https://www.ufrgs.br/telessauders/perguntas/corticoide-oral/. Accessed 25 Jun 2020.

